# Interrelation between defensive mechanisms and coping strategies in psychiatry trainees in Romania: a multicenter study

**DOI:** 10.1186/s12991-020-00307-1

**Published:** 2020-09-27

**Authors:** Anca-Livia Panfil, Mirela Frandes, Aurel Nirestean, Marinela Hurmuz, Diana Lungeanu, Madalina Cristanovici, Laura Lemeti, Andra Isac, Ion Papava, Cristina Bredicean

**Affiliations:** 1 Doctoral School, Department of Psychiatry, “George Emil Palade” University of Medicine, Pharmacy, Science and Technology, Târgu Mureş, Romania; 2 Department of Functional Sciences-Medical Informatics and Biostatistics, “Victor Babes” University of Medicine and Pharmacy, Eftimie Murgu Sq. no. 2, 300041 Timisoara, Romania; 3 Department of General Psychiatry, Psychiatric Clinic II, Târgu Mureș, Romania; 4 Consultant Psychiatrist, CNWL NHS Foundation Trust, Milton Keynes, UK; 5 Child and Adolescent Psychiatry and Addictions Clinic, Children’s Emergency Hospital, Cluj-Napoca, Romania; 6 Department of Neuroscience-Psychiatry, “Victor Babes” University of Medicine and Pharmacy, Timisoara, Romania

**Keywords:** Structural equation modeling, Defense mechanisms, Coping behavior

## Abstract

**Background:**

The challenges faced by professionals when working in the field of psychiatry require the development of adequate defensive and coping mechanisms. This study aimed to explore both coping strategies and defense mechanisms and their relationship in psychiatry trainees in Romania.

**Methods:**

A cross-sectional study was conducted to determine and evaluate both defensive and coping mechanisms of Romanian psychiatry trainees. Defensive Style Questionnaire-60 and COPE scale were applied to psychiatry trainees from five training centers in Romania. By applying structural equation modeling, models that presumed the existence of relationships between coping strategies and defensive mechanisms were analyzed.

**Results:**

Superior defense mechanisms and task-oriented coping strategies were the commonly used approaches by psychiatry trainees. Furthermore, significantly consistent correlations (ranging from 0.2 to 0.5) between adaptive defense mechanisms and coping strategies focused on the problem or emotion were shown. Similarly, avoidant coping strategies correlated with non-adaptive defense mechanisms (correlations between 0.3 and 0.5). Our model presented good fit indices (*X*^2^(34) = 64.324, *p* < 0.001; GFI = 0.93; root mean square error = 0.08). Moreover, the results indicated a weak association between the two types of adaptive processes (*r* = 0.07, *p* < 0.001).

**Conclusion:**

Psychiatry trainees present a profile based on two independent groups of adaptation processes, namely, adaptive defenses and problem-oriented coping scales and non-adaptive defenses and avoidant coping scales.

## Background

The relationship between coping and defense mechanisms is controversial [1–3]. The term “defense mechanisms” was defined in 1926 by Freud in an attempt to explain how individuals manage stress [4]. Traditionally, defense mechanisms are patterns of relatively involuntary responses [5] to external or internal factors and involve feelings, thoughts, and behaviors [6]. Coping mechanisms are conscious and purposeful processes [2] and based on cognition [7]. The concept of defense was difficult to differentiate from coping, as the two were frequently misused or their definitions overlapped [2]. Lazarus suggested that coping and defense mechanisms must be studied together and therefore coping should not be limited to deliberate and conscious processes [8]. The relationship between coping and defense mechanisms might explain the debate on these concepts and misuse of terminology and confusion throughout the literature.

Coping and defense mechanisms have been analyzed with respect to different symptoms and disorders, such as depression, anxiety, or personality disorders [9]. The findings showed that coping and defense mechanisms tend to have a certain pattern with these disorders and certain mechanisms, such as avoidant coping, might increase future symptoms [10]. This is related to the vulnerability-stress psychopathology model that outlines two underlying components [11]. The internal component, vulnerability, comprises all mechanisms and processes that arise and are non-adaptive. The external component, stress, is based on life events [12]. The two influence each other, creating a threshold for disorders to arise [13]. The types of coping and defense mechanisms used may contribute to vulnerability, as previously mentioned, or can be protective factors. In this regard, Perry and Carver et al. proposed a hierarchy of seven levels of defense mechanisms [14] and four strategies of coping [15].

Evaluation of medical doctors’ coping and defenses has not been frequently considered, although numerous studies have reported high levels of burnout in this profession [16, 17], along with high rates of depression and suicide ideation among resident physicians and medical students [18, 19]. Psychiatry is a stressful medical field [20], and psychiatrists are prone to burnout and even suicide [21, 22]. Younger psychiatrists tend to be more stressed than older ones [23], and women are more stressed than men [24]. A major stress factor is patients’ suicide, with younger psychiatrists being more affected than their senior colleagues [21, 23]. Other sources of stress are negative attitudes of patients and caregivers, administrative and management shortcomings, overload, and poor resources [25, 26]. Although emerging evidence has shown that debriefing after a traumatic event may not be of help for all patients [27, 28] or in all circumstances [29], it remains the standard procedure in many clinical settings. Hearing patients’ traumatic history can also cause stress among clinicians [30]. Regarding the influence of stress exposure on coping and defense mechanisms, some argue that it can either increase the risk of developing mental health issues [31–33] or facilitate the development of more efficient coping and defense mechanisms in certain circumstances [34].

We conducted an initial pilot study to explore both the coping and defense mechanisms of psychiatry trainees in Romania [35]. Based on this study, we explored the possible relationship between coping strategies and defense mechanisms and their implications in clinical practice. In Romania, twelve medical centers are in charge of conducting residency programs. The duration of training was 5 years. All centers have the same curriculum in their psychiatric training program. Psychiatry trainees work in psychiatry university hospitals with rotations in the general hospital. They work 35 h per week, and a minimum of 24 h of on-call duty per month is also required. They are assigned to a coordinator who supervises their performance. They are also assigned to a clinical supervisor responsible for clinical activity. The main focus of psychiatrists’ training is on patient care, with no specific attention paid to psychiatrists’ personal development. Furthermore, no personal guidance is included in the training program. Moreover, there is no specific training in managing personal difficulties and improving self-care and self-development. Consequently, psychiatry residents are more vulnerable to burnout, mental health issues, or psychiatric disorders.

This study aimed to explore coping and defense mechanisms and their relationships in psychiatry trainees in Romania. With a better understanding of these mechanisms, resources can be redirected to the most effective interventions in regard to developing and maintaining protective factors against psychopathology. The exploration of the relationship between coping and defense mechanisms can offer practical guidance for future interventions.

## Methods

### Study sample

Romanian psychiatry trainees from five different training centers and different regions in Romania (Bucharest, Cluj-Napoca, Timisoara, Târgu Mures, and Sibiu) were invited to participate. Questionnaires were distributed with the help of the Romanian Psychiatric Trainees Association’s national network. Local coordinators were involved in distributing and collecting the on-paper questionnaires based on their contact with local trainees.

Data collection was conducted between March 2016 and September 2016. The inclusion criteria were enrollment in a psychiatry training program in Romania, Romanian nationality, and agreement to complete the questionnaires. All participants were informed about the purpose of the study and confidentiality of the collected data. No remuneration was offered.

We estimated a total of 604 active psychiatric trainees at the time of the study, 398 in the centers where we conducted the research. We did not consider the possible dropouts from the training program, changes in specialty, or migration of trainees, which is one of the highest in Europe [36]. A total of 133 questionnaires were collected. By selecting the ones that had a maximum of three questions with no response, 112 questionnaires were included in the study. In the case of missing answers, the response was completed with the corresponding mean values.

### Measurements

Participants were asked to fill in two self-assessment questionnaires that evaluate their coping styles: COPE [15], a scale with 60 questions that explored 15 coping mechanisms and defense mechanisms, and Defensive Style Questionnaire (DSQ)-60 [37], a self-report measure with 60 questions that explored 30 defense mechanisms. We selected these scales because they offer some advantages in the assessment of adaptive mechanisms. COPE is the most used scale in this field, and DSQ-60 assesses the defense mechanisms compatible with those included in the Diagnostic and Statistical Manual of Mental Disorders, 4th edition (DSM-IV) [38]. The two self-assessment questionnaires were translated, validated, and adapted for the Romanian population [39, 40].

### Assessing coping strategies

The COPE scale comprised 60 items graded on a Likert-type scale from 1 to 4, where 1 indicates “I usually don’t do this” and 4 indicates “I often do this.” The four coping strategies [15] are as follows:

- Emotion-focused coping: positive interpretation and growth, restraint, and acceptance.
- Problem-focused coping: planning, active approach, and deletion of concurrent activities.
- Social support coping: social instrumental support, use of social–emotional support, and expression of feelings.
- Avoidant coping: denial and mental and behavioral deactivation.

The highest score recorded in these four coping strategies was considered in this study as the dominant and most representative of the patients investigated.

### Assessment of defensive mechanisms

The DSQ-60 is a self-report measure to assess the 30 defense mechanisms included in the DSM-IV [38]. The questionnaire has 60 items that are evaluated using a 9-point Likert-type scale (1, not at all applicable to me; 9, completely applicable to me). The seven levels of defense mechanisms [14, 39] are as follows:

- Action: help rejecting, complaining, acting out, and passive aggression.
- Major image distortion: projective identification, splitting of others, and splitting of the self.
- Refusal to take responsibility: fantasy, rationalization, projection, and denial.
- Minor distortion of the image: devaluation of other, devaluation of self, self-idealization, and the idealization of the other, omnipotence.
- Neurotic: displacement, reaction formation, dissociation, and repression.
- Obsessive: isolation of affect, intellectualization, and undoing.
- Adaptive: sublimation, suppression, self-assertion, self-observation, humor, anticipation, altruism, and affiliation.

### Statistical analysis

Data are presented as median (interquartile range) for continuous variables with non-Gaussian distribution or absolute frequency (percentage) for categorical variables. Continuous variable distributions were tested for normality using the Kolmogorov–Smirnov’s test and equality of variances using Levene’s test.

To assess the significance of the differences between groups, Student’s t-test (means, Gaussian populations), Mann–Whitney *U* test (median, non-Gaussian populations), and Pearson chi-square or Fisher’s exact test (proportions) were used. Using structural equation modeling, we studied models that assumed the existence of relationships between coping and defensive mechanisms.

We applied structural equation modeling (SEM) to overcome the problem of multiple hypotheses testing. The SEM approach is more appropriate when analyzing noncasual relationships, such as relationships between coping and defenses. The SEM approach does not assume that one variable is a predictor for other variables. Moreover, the SEM has the convenience of allowing comparisons between complex models, such as models that assumed the existence of relationships between coping and defenses and models that assumed independence between these constructs. More precisely, we applied the SEM approach with the maximum likelihood estimation method. Moreover, we reported fit indices that are least influenced by the estimation method [goodness-of-fit index (GFI) ] or sample size [root mean square error of approximation (RMSEA)]. For comparison purposes, we also reported the chi-square index and comparative fit index (CFI). Acceptable fit is indicated by values < 0.08 for RMSEA and > 0.90 for CFI. We considered that the two structural models were different when Δ*X*^2^ was statistically significant, and the difference between the CFI of the two models was > 0.01.

Data were analyzed using SPSS version 17 software (SPSS Inc., Chicago, IL, USA) and R software packages (version 3.3) for statistical computing. A *p*-value < 0.05 was considered the threshold for statistical significance. A confidence level of 0.95 was considered for estimating intervals.

## Results

### Sociodemographic characteristics

The sociodemographic characteristics of the sample are presented in Table 1. There were 112 participants who agreed to participate in the study. Participants were recruited from different training centers and regions in Romania: Bucharest, Cluj-Napoca, Timisoara, Târgu Mures, and Sibiu. The average age of the lot was 27 (26–30) years, and there were 71 women (63.4%), with an average period of work in the field of 7 (3–42) months. Most residents were in the first year (38.4%), while 27.2% and 20.2% of the residents were in the fourth and third years, respectively.

**Table 1:** Sociodemographic characteristics of the participants

Number of participants	112
Age (years)^a^	27.0 (26.0–30.0)
Gender (female)^b^	71 (63.4%)
Citizenship^b^	
Romanian	110 (98.2%)
Other	2(1.8%)
Education^b^	
Bachelor's degree	108 (96.5%)
Master's degree	3 (2.6%)
PhD	1 (0.9%)
Year of residency^a^	2 (1–4)
Experience in psychiatry (months)^a^	24 (7–41)
Civil status^b^	
Married	32 (28.6%)
Unmarried	70 (62.5%)
Single	4 (3.6%)
Cohabiting partnership	6 (5.3%)
Number of children^a^	
None	94 (83.9%)
One	14 (12.5%)
Two	4 (3.6%)
Working period (months)^a^	7 (3–42)
Religion^b^	
Orthodox	86 (76.8%)
Catholic	5 (4.5%)
Greco-catholic	1 (0.9%)
Reformed	3 (2.7%)
Agnostic	2 (1.8%)
Independent	1 (0.9%)
Atheist	5 (4.4%)
Undeclared	9 (8.0%)

^a^Continuous variables (with non-Gaussian distribution) are indicated by their median (interquartile range-IQR)

^b^Categorical variables are presented by absolute frequency and percentage in the sample

More than half of the participants were unmarried (62.5%) and do not have children (83.9%). We also observed that more than half of the participants had a Christian orthodox religion (76.8%).

Table 2 presents the description of all coping strategies with problem-focused and emotion-based coping styles with a median of 36 (33–40) and 51 (47–56), respectively.

**Table 2:** Description of coping strategies

Coping style	Median	25%	75%	Minimum	Maximum
Problem-focused	36	33	40	22	48
Emotion-focused	51	47	56	31	74
Social support	32.5	29	37	17	46
Avoidant	27	24	31	17	44

The dominant coping style was “problem-focused” for almost half of the participants (46.1%). The emotion-focused coping style was the dominant coping style for 26.3% of participants, while, for 25% of participants, the dominant coping style was social support. Moreover, 2.6% of the participants used avoidance-type coping as their dominant coping style.

Table 3 shows a description of the defensive mechanisms for both adaptive and non-adaptive strategies. Superior adaptation has a median of 95 (86–105), while mental inhibition and minor distortion presented a median of 52.5 (41–63) and 20 (15–26.5), respectively.

**Table 3:** Description of defensive mechanisms

Defensive mechanism	Median	25%	75%	Minimum	Maximum
Superior adaptation	95	86	105	61	129
Disavowal	23	19.5	28	10	52
Mental inhibition	52.5	41	63	28	92
Major distortion	21	14	28	8	51
Minor distortion	20	15	26.5	8	43
Action	27.5	22	36	10	59

We observed significantly consistent correlations (ranging from 0.2 to 0.5) between adaptive defense mechanisms and coping focused on the problem, emotion, or seeking social support. First, a significant positive correlation between superior adaptation and problem-focused coping style (Spearman’s *r* = 0.381, *p* < 0.01) was observed. We also found a significant positive correlation between superior adaptation and emotion-focused coping (Spearman’s *r* = 0.266, *p* < 0.01) and social support coping (Spearman’s *r* = 0.255, *p* < 0.01). Furthermore, the superior adaptation mechanism was negatively correlated with avoidant coping strategy (Spearman’s *r* = − 0.091, *p* = 0.338) (Table 4).

**Table 4:** Correlations between defensive mechanisms and coping strategies

Parameters	Problem-focused	Emotion-focused	Social support	Avoidant
Superior adaptation	0.381**	0.266**	0.255**	− 0.091
Disavowal	0.027	0.059	0.163	0.191*
Mental inhibition	0.035	0.109	0.143	0.415**
Major distortion	− 0.158	− 0.112	0.222*	0.420**
Minor distortion	0.057	0.098	0.202*	0.277**
Action	− 0.070	− 0.069	0.198*	0.410**

^*^Significant correlation at 0.05 level

^**^ Significant correlation at 0.01 level

The avoidant coping strategies were correlated with defense styles that are not adaptive (correlations between 0.2 and 0.5). More precisely, we observed a significant positive correlation between major distortion and avoidant coping style (Spearman’s *r* = 0.420, *p* < 0.001), between mental inhibition and avoidant coping style (Spearman’s *r* = 0.415, *p* < 0.001), and between action and avoidant coping style (Spearman’s *r* = 0.410, *p* < 0.001).

Another significant positive correlation was found between major distortion and social support coping mechanisms (Spearman’s *r* = 0.222, *p* = 0.019) and minor distortion and social support coping mechanisms (Spearman’s *r* = 0.202, *p* = 0.033). In contrast, disavowal and mental inhibition were not significantly correlated with social support coping style (Spearman’s *r* = 0.163, *p* = 0.086; Spearman’s *r* = 0.143, *p* = 0.134, respectively). Moreover, disavowal, mental inhibition, and major and minor distortions were significantly correlated neither with problem-focused coping nor with emotion-focused coping.

**Fig. 1:**
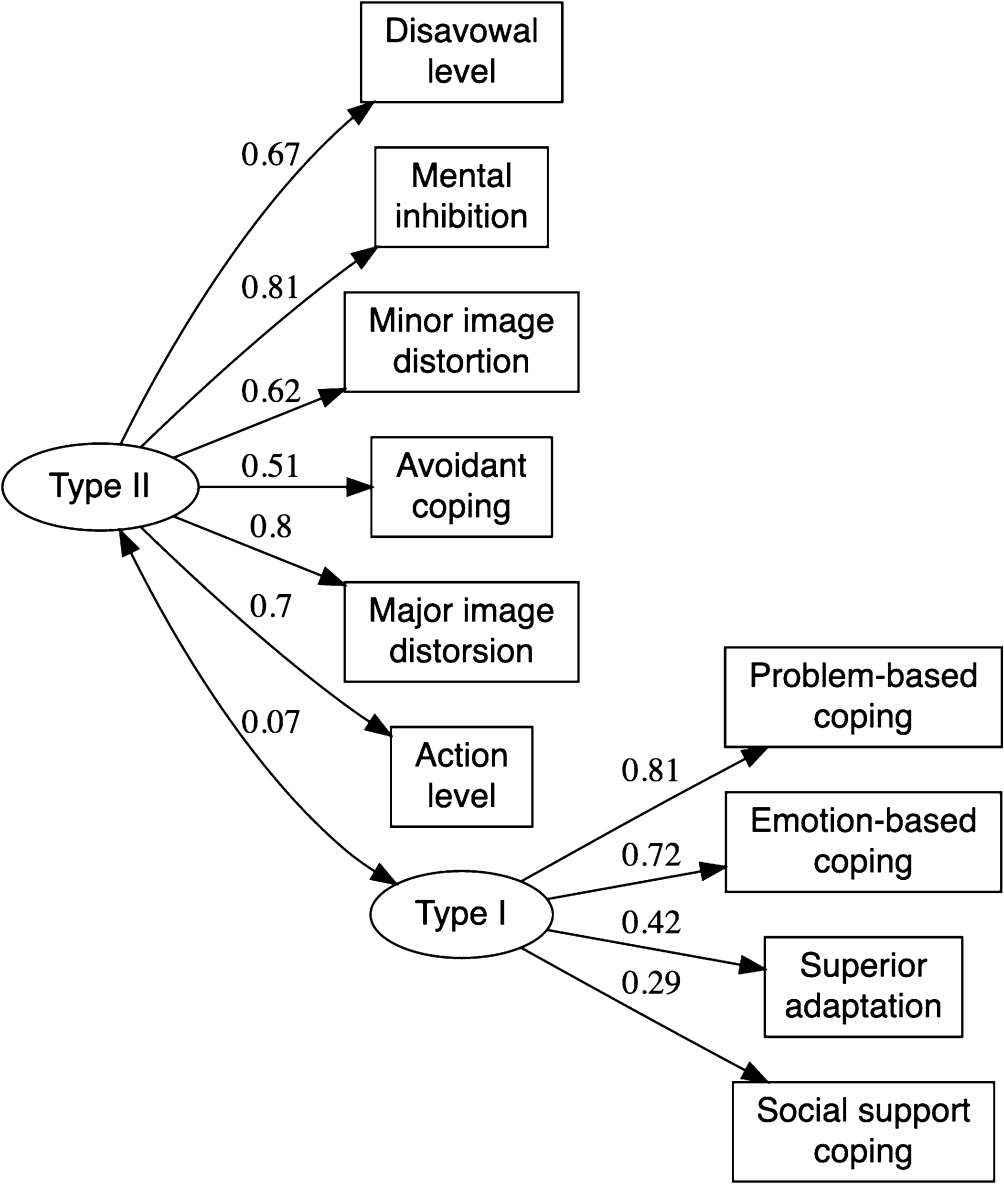
Graphical representation of the model showing the two types of the adaptation process, corresponding to different psychiatry residents’ profiles

We grouped coping and defense mechanisms into two types of adaptive processes: one type of adaptive process includes mature (or well adaptive) defenses and all forms of active coping (focused on the problem, focused on emotion, or focused on seeking social support); the other type includes non-adaptive defense mechanisms and avoidant coping (Fig. 1).

Our model presented GFIs (*X*^2^(34) = 64.324, *p* < 0.001, GFI = 0.93, RMSE = 0.08). Moreover, the results indicated a very weak association (*r* = 0.07, *p* < 0.001) between the two types of adaptive processes. Correlation values close to zero were found between avoidant coping and adaptive defense mechanisms and between non-avoidant coping (problem-focused, emotion-focused) and non-adaptive defenses.

## Discussion

This study aimed to investigate the relationships between coping strategies and defense mechanisms in psychiatry trainees. We found that the dominant coping style was a problem-focused strategy, followed by emotion-focused coping and social support styles. The dominant defense mechanism was superior adaptation, followed by mental inhibition and minor image distortion. We also observed a positive and significant correlation between superior adaptation and problem-focused coping styles and between superior adaptation and emotion-focused coping. The avoidant coping strategies correlated with defense styles that are not adaptive, with major distortion. Another significant positive correlation was found between major distortion and social support coping strategies. Major distortion was negatively correlated with emotion-focused coping. Based on these findings, we observed that these adaptive processes are grouped into two different categories, corresponding to different psychiatry residents’ profiles.

The gender shift in the medical profession [41, 42] may explain why more than half of our participants were women (approximately 63.4%). Factors such as female sex [43, 44] and unmarried (62.5%) status [45] are considered by many studies as factors of vulnerability for mental health, while having no children (83.9%) protects the subjects from the potential stress of parenthood [45]. This can also suggest a life trajectory in the medical profession that involves many years dedicated to professional development and less time dedicated to personal life [46].

The residents’ experience in psychiatry was between 6 and 48 months, allowing the supposition that the job involved in changing the structure of coping and defense strategies was limited [47–49]. Trainees used mature defense mechanisms. Some studies found that this is less common in a younger age group and more common in the > 40 years of age group [50]. Younger psychiatrists tend to use better defense strategies but are more likely to feel more stressed than their older colleagues [51]. The general population seems to have more mature strategies with age [52]. The very nature of work undertaken by psychiatrists can likely be a cause of vulnerability to stress, generating changes in defense and coping [23, 51, 53]. Stressful events can induce regression to inferior defensive styles and coping [54–56]. This population might have an atypical age-related evolution in the use of coping and defense mechanisms that require further investigation.

In several studies, problem-focused coping style has been associated with positive emotional well-being in different organizations [57–60], while emotion-based coping may be a factor in the emotional strain of a person [59, 60]. Coping efficacy depends not only on the type of coping strategy used but also on many factors, such as personal and organizational [61–63]. Although problem-focused coping is reported to be superior to emotion-focused coping, both functions are usually used together in the same process, in different degrees [64]. Affect and cognition are not separated and usually used together in executive functions, such as decision-making. This may explain the correlation of both coping strategies with superior adaptation.

Management should consider these findings when planning psychological interventions for personnel. Psychological support and personal development should be encouraged, especially when facing stressful events, such as patient suicide, so that the adaptive response to stress is maintained career-long.

The results also suggest that coping and defense mechanisms could be grouped into two types of adaptive processes. Starting from these observations, we tested a model that assumed the existence of two types of adaptive strategies. We used the composite score for each of Carver’s four types of coping [15] and the composite score for each of Perry’s seven defense levels [14].

Based on the correlations found, we can suggest that adaptive defense mechanisms imply coping focused on the problem, emotion, or seeking social support with a stronger probability of problem-focused coping style, while excluding avoidant coping strategies. The major distortion mechanism has a good probability of implying avoidant coping style or social support coping mechanisms, but not an emotion-focused coping strategy. This study sustains the grouping of the adaptive processes proposed by Maricutoiu and Crasovan [1]. Taking into consideration these findings, a new way of evaluating coping and defense mechanisms, paired together, as adaptative processes, can be developed and knowledge may be extended from one to another.

### Strengths and limitations of the current study

This study is one of the few conducted on this specific population. We recruited medical trainees from different geographic regions and training centers in Romania. We used well-known, adapted, and validated instruments.

The most important limitation of our study is that the sample was selected based on the response to an invitation of participation made by local coordinators to their circle of colleagues, which can raise the problem of selection bias, excluding professionals outside this circle. By making an online assessment, we could have avoided this problem. We believe that, because of the personal content of the questionnaires, a more friendly and personal approach was more culturally appropriate.

## Conclusion

Problem-focused and emotion-based coping styles were the best-represented coping styles within the sample. Avoidant coping was the least used coping style. Superior adaptation was the best-represented defense strategy, followed by mental inhibition. The least used defense strategy was major distortion. Our results support the two hypothesized relationships with the associations between adaptive defenses and problem-oriented coping and associations between non-adaptive defenses and avoidant coping. This grouping of adaptive processes is a newly emerging idea that needs further studies.

## Data Availability

The datasets used and/or analyzed during the current study are available from the corresponding author on reasonable request.

## Abbreviations

DSQ-60: Defense Style Questionnaire-60
COPE: Coping inventory
SEM: Structural equation modeling
GFI: Goodness-of-Fit Index
RMSE: Root-mean-square error of approximation
IQR: Interquartile range
SPSS: Statistical Package for the Social Sciences software
